# Tuberculous Otitis Media Leading to Sequentialib Bilateral Facial Nerve Paralysis

**Published:** 2015-05

**Authors:** Nitin Gupta, Arjun Dass, Neha Goel, Sandeep Tiwari

**Affiliations:** 1 *Department of Otorhinolaryngology, Govt Medical College and Hospital*, *Sector-32, Chandigarh, India. *

**Keywords:** Abdominal tuberculosis, Bilateral facial nerve paralysis, Tuberculous otitis media

## Abstract

**Introduction:**

Tuberculous otitis media (TOM) is an uncommon, insidious, and frequently misdiagnosed form of tuberculosis (TB). In particular, TOM is usually secondary to direct transmission from adjacent organs, while the primary form has been rarely reported. The main aim of treatment is to start the patient on an antitubercular regime and early surgical intervention to decompress the facial nerve if involved.

**Case Report:**

The case report of a twenty year-old male with bilateral tuberculous otitis media, who presented himself with fever followed by sequential bilateral facial nerve paralysis, bilateral profound hearing loss, and abdominal tuberculosis leading to intestinal perforation, is presented. To the best available knowledge and after researching literature, no such case depicting the extensive otological complications of tuberculosis has been reported till date.

**Conclusion:**

Tuberculosis of the ear is a rare entity and in most cases the clinical features resemble that of chronic otitis media. The diagnosis is often delayed due to varied clinical presentations and this can lead to irreversible complications. Early diagnosis is essential for prompt administration of antitubercular therapy and to prevent complications.

## Introduction

Tuberculosis (TB) remains a major health problem in the developing world, where almost over eight million new cases of TB are diagnosed annually (1). Pulmonary TB is the most common clinical presentation, while 15–20% of cases manifest as extrapulmonary or disseminated TB. The most frequent sites of extrapulmonary TB are the lymph nodes (48.9%), pleura (25.5%), skeleton (22.7%), genitourinary tract (5.7%), and meninges (5%) (2). However, TB can also manifest itself in the eye, brain, pericardium, peritoneum, abdominal organs, skin, and other sites. 

Tuberculous otitis media (TOM) is an uncommon, insidious, and frequently misdiagnosed form of TB (3). In particular, TOM is usually secondary to direct transmission from adjacent organs (i.e. the lungs, larynx, pharynx, and nose), while the primary form has been rarely reported. Half of the cases have no other evidence of present or past infection, and its diagnosis is often delayed due to its rarity or its usually indolent course (4,5). Patients can classically be presented with multiple perforations of the tympanic membrane, pale granulations, severe mixed hearing loss, and facial palsy. The main aim of treatment is to start the patient on an antitubercular regime and early surgical intervention to decompress the facial nerve if involved. 

## Case Report

K.C a twenty year-old patient of Indian nationality was presented to the ENT department for evaluation of an 8 month history of bilateral ear discharge with progressive hearing loss associated with a one day history of fever. The discharge was yellowish in colour, foul smelling, painless, and non blood stained. 

The hearing loss was progressive and bilateral; and it interfered with the normal communication of the patient. There was no history of associated vertigo or facial nerve paralysis. Fever had been present for one day, but it was not associated with chills and rigors and was relieved with medication. There was no previous history of hypertension, diabetes, or tuberculosis. 

**Fig 1:**
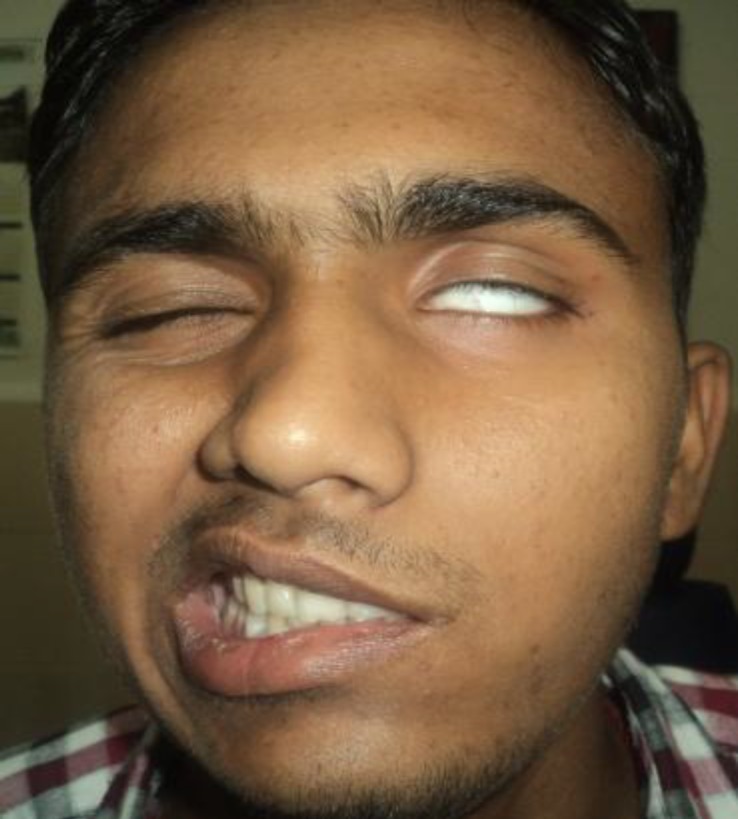
Left sided facial palsy, grade 5

Within a day of being admitted to the hospital for fever evaluation, the patient developed left sided facial nerve paralysis, which on examination was identified as Grade V using the House Brackmann grading system (Fig.1). 

Otoscopic examination of the left ear revealed total perforation with the presence of a purulent discharge. The right ear also showed the presence of a similar discharge with total perforation. His other systemic examination findings were normal, except for anaemic conjunctivae. A provisional diagnosis of bilateral unsafe chronic suppurative otitis media with left facial nerve paralysis was made. Pure tone audiometry showed bilateral profound hearing loss (98 dB bilaterally).

**Fig 2:**
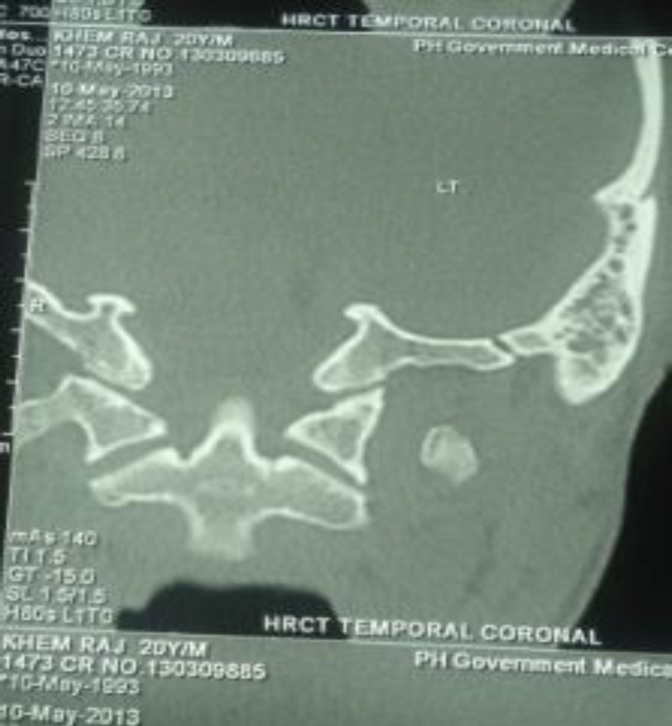
HRCT temporal bone showing mastoid destruction with haziness of cells

Laboratory values included a white blood cell count of 42,800/mm^3^ (86.5% segmented neutrophils, 7.9% lymphocytes, 5% monocytes, 0.5% eosinophils, and 0.1% basophils), a haemoglobin count of 9.9 g/dL, an erythrocyte sedimentation rate ofwas 93mm in the 1^st^ hour, and a platelet count of 1,85,000/mm^3^. The fasting blood sugar concentration was 84 mg/dl. Blood and urine cultures as well as sputum examination were negative. Serology for human immunodeficiency virus was negative. Chest X-ray was normal. High resolution CT scan of the temporal bone revealed destruction of the mastoid cavity along with soft tissue attenuation (Fig. 2). 

**Fig 3:**
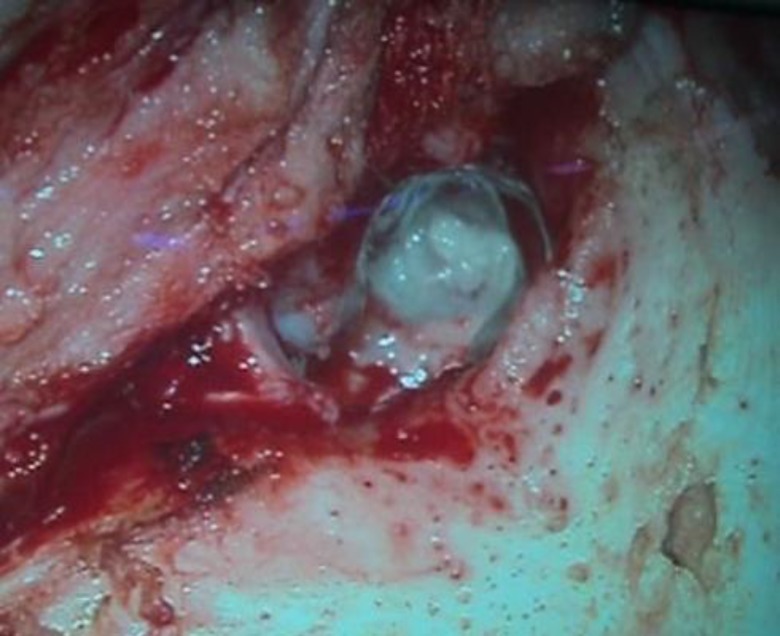
Examination under microscope showing left middle ear with sticky white discharge

The patient was started on intravenous amoxyclav (1000mg of amoxicillin and potassium clauvlate with 200 mg of TDS) along with a course of oral deflazacort (60 mg started on the day of the development of facial paralysis, which was tapered off gradually). An emergent surgical procedure of mastoid exploration was scheduled to improve facial nerve paralysis within the 48 hours that it was developing. Under general anaesthesia, examination under the microscope revealed the presence of a sticky white cheesy discharge in the middle ear, which could not be suctioned, and was adherent to the promontory mucosa (Fig.3). 

**Fig 4:**
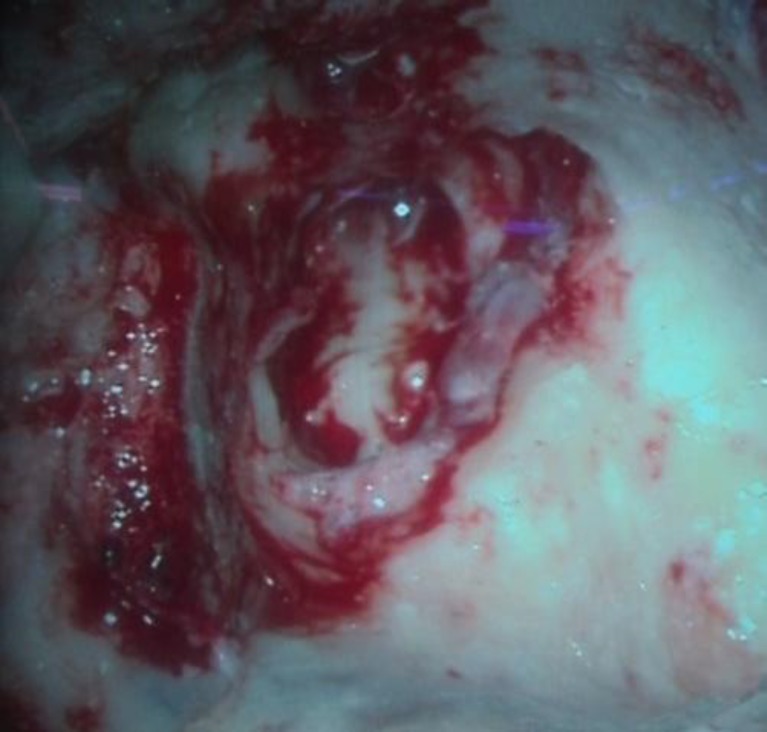
Left ear intraoperative picture showing haematoma of the horizonal part of the facial nerve

A significant amount of granulation tissue was removed from the mastoid antrum, aditus, attic, and middle ear by canal wall down mastoidectomy. During the operation, edematous facial nerve in the horizontal and vertical segment was identified. There were granulations and a haematoma in the horizontal segment of the facial nerve (Fig.4) and partial dehiscence of the facial fallopian canal in the vertical segment. 

**Fig 5:**
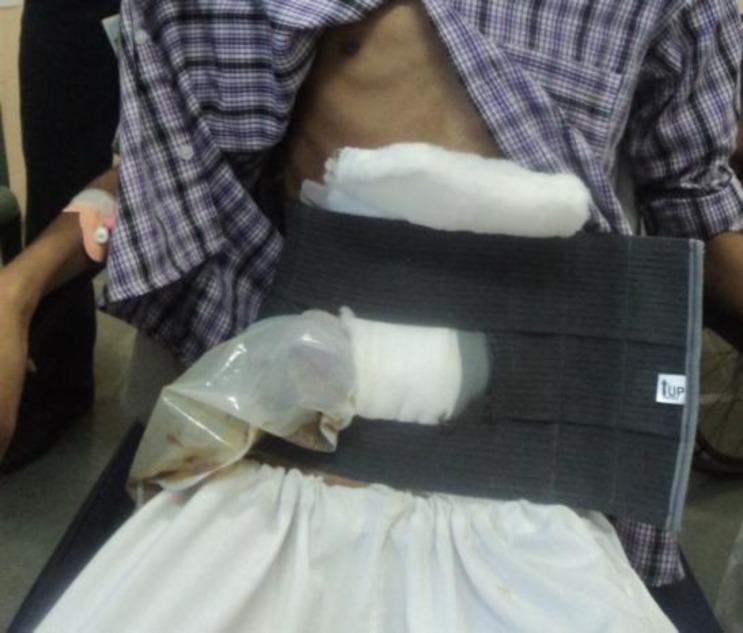
Abdomen with colostomy bag

Facial nerve decompression was performed by removing the bone of the fallopian canal and incising the sheath of the facial nerve in the vertical segment to resolve facial nerve paralysis. A small fistula (about 1 mm) was present in the superior semicircular canal. All the ossicles were engulfed by granulation tissue. Remnant of the handle of the malleus, partly necrosed incus, and stapes suprastructure, and footplate could be identified. Malleus and incus were removed. Osteitic changes were present in the mastoid antrum and semicircular canals. Meatoplasty was performed followed by the sealing of the mastoid cavity by using a temporalis fascia graft. On the first postoperative day of the surgical procedure, the patient developed acute abdominal pain with guarding and rigidity of whole abdomen. Abdominal X-ray revealed an intestinal perforation for which the patient underwent exploratory laprotomy on post-operative day two. There was presence of two litres of seropurulent fluid in the abdominal cavity with feculent smell, matted gut loops, and pus flakes. Approximately 30 cm from the ileocaecal junction, a single ileal perforation was present. This was repaired and patient was put on a colostomy tube (Fig. 5).

**Fig 6:**
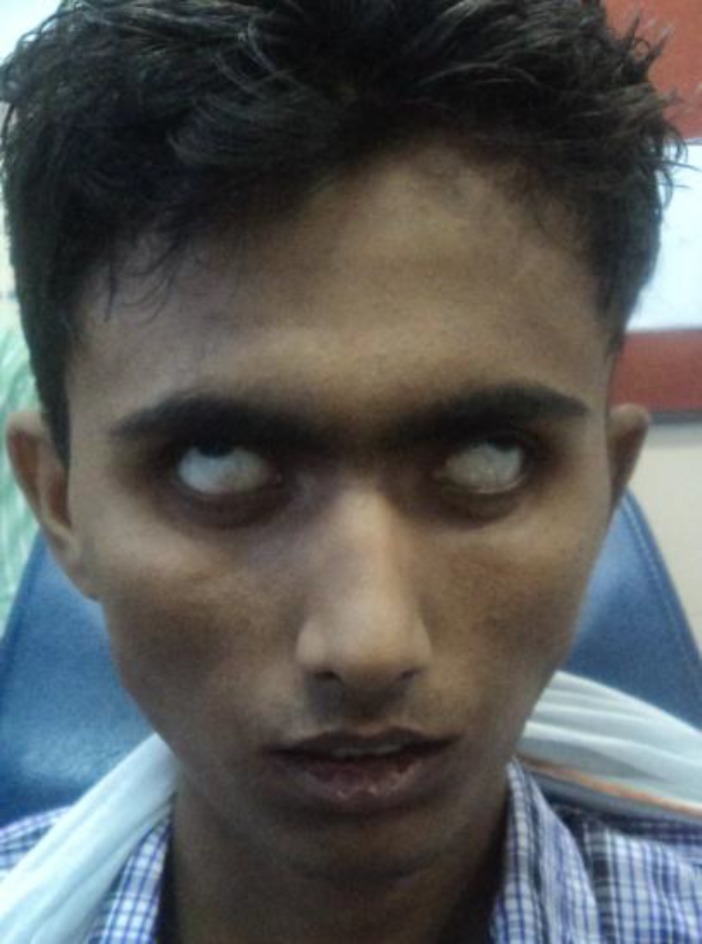
Post operative day 7, patient showing bilateral facial palsy, grade 5.

Following laprotomy, the patient developed right sided facial nerve paralysis of House Brackmann grade V on postoperative day 7 (of left ear surgery) (Fig. 6).

**Fig 7:**
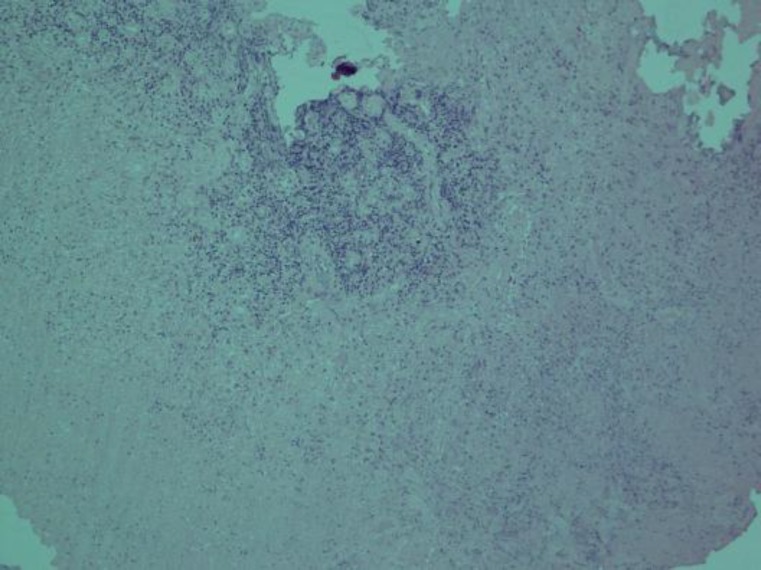
Histopathological examination showing fibrocollagenic tissue with inflammatory granulation tissue formation, epitheliod cell granulomas with Langhan’s type giant cells, and areas of caseous necrosis

The tissue sent for histopathology from the left middle ear and abdominal cavity showed presence of a fibrocollagenic tissue with inflammatory granulation tissue formation, epitheliod cell granulomas with Langhan’s type giant cells, and areas of caseous necrosis. Definite acid-fast bacilli were identified under special staining (Fig.7). A diagnosis of tuberculous otitis media (TOM) along with abdominal tuberculosis was made. PPD test was not done as this is a nonspecific test and the biopsy report was positive for tuberculosis.

The patient was then started on a 12-month course of a four-drug anti-tuberculosis treatment (ATT) with oral rifampicin, isoniazid, ethambutol, and pyrazinamide. The patient was then kept on regular follow up.

**Fig 8:**
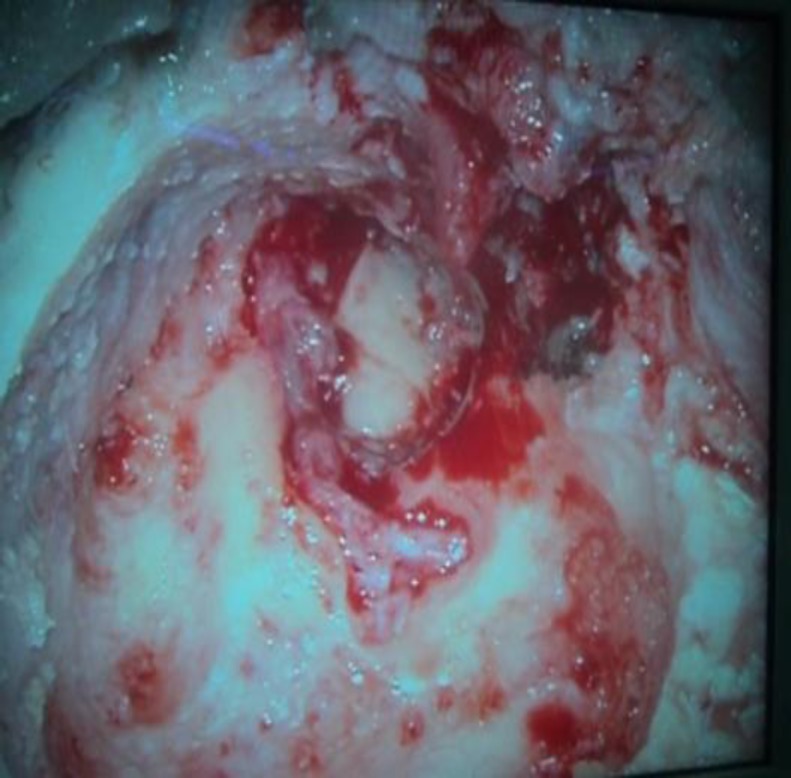
Right ear intra-operative picture showing facial nerve covered in granulation tissue

 After 2.5 months of taking ATT, the patient was taken up for right ear surgery as there was no significant improvement in facial nerve functions. Intraoperative findings included pale granulation tissue in the antrum, aditus, and attic, extending to involve the anterior epitympanum and sinus tympani area, which were removed. The malleus was absent and the long process of the incus was necrosed. The stapes suprastructure was present but engulfed in granulation tissue. The facial nerve was covered in granulation tissue (Fig.8) and was completely decompressed in both the vertical and horizontal segments.

**Fig 9:**
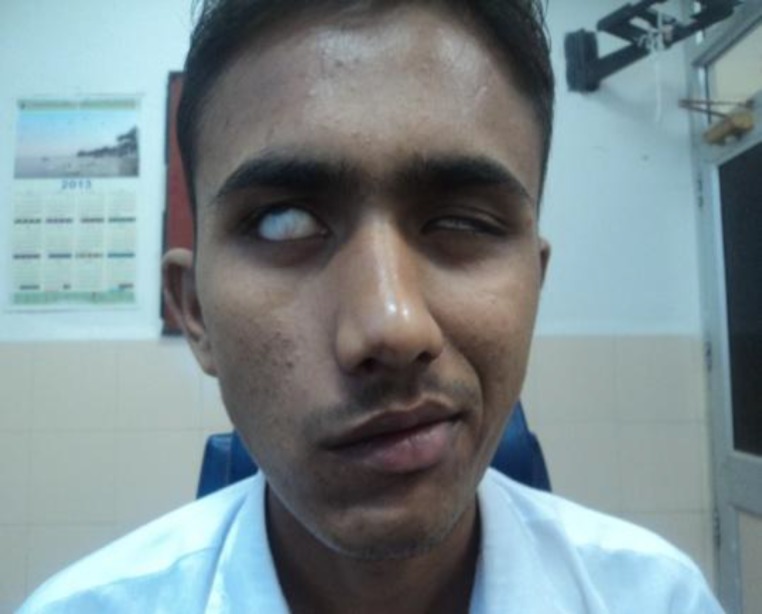
4 month follow up showing improvement in left facial nerve functions

Post-operatively and during the course of the hospital stay, the patient showed improvement in facial nerve functions bilaterally. Four months after the first surgery, complete closure of left eye with maximal effort was seen (Fig. 9). Both the mastoid cavities were healthy and had no discharge. The abdominal wound also healed completely and had a healthy scar mark. The patient has been in follow-up for the last 8 months and is still on antitubercular therapy.

## Discussion

In a series of 323 cases of extrapulmonary TB, ENT localization was seen in 23.2% if cases, of which 94.1% were in the cervical lymph nodes, 4.33% in the larynx, 0.62% in the tonsil, 0.31% in the oral cavity, 0.31% in the middle ear, and 0.31% in the nose (6). Two smaller studies from India reported roughly similar findings, but included rare cases of TB of the cervical spine, parotid, temporomandibular joint, and a retropharyn- geal abscess (7,8).

TOM was first reported in 1853, and the organism was first identified in an ear discharge in 1883 (9,10). Although the pathogenesis of TOM is still controversial, three mechanisms explaining middle ear tuberculosis infection have been postulated: aspiration of mucus through the auditory tube, hematogenous transmission from other tuberculosis foci, and direct implantation through the external auditory canal with tympanic membrane perforation (9,10,11). In this case, the source of focus was presumably in the gut. TOM is a rare cause of chronic suppurative infection of the middle ear, ranging from 0.05 to 0.9% of chronic otitis media TB (5). Because of similar symptoms and signs, its differential diagnosis from non-TB chronic otitis media may be quite difficult (9). 

Of 52 patients with TOM in South Korea (12), the main symptom was otorrhea with mucopurulent discharge, 13.5% reported otalgia, 28.8% tinnitus, and 13.5% experienced vertigo, probably as a result of the destruction of the semicircular canals. Peripheral facial palsy was found in 9.6% of patients, which is higher than patients with otitis media in general, thus raising suspicion of TOM, especially in the absence of cholesteatoma. Facial palsy was associated with a shorter duration of symptoms (mean 9.4 months versus 106.3 months). Conductive hearing loss occurred in 56.6% of patients and a mixed hearing loss in the remainder. 

Otoscopic examination revealed perforations of the tympanic membrane or adhesions. In addition, the clinical manifestations of this disease are not always in accordance with the description of the classical triad of painless otorrhea, multiple tympanic perforations, and facial palsy TB (12, 13). Therefore, initial anti-tuberculosis treatment is often delayed. Complications occur mostly when diagnosis is delayed, and include facial paralysis, labyrinthitis, meningitis, and subperiosteal abscesses, among others (14). Facial paralysis may be present in 15-40% of TOM cases, more frequently in children (9,14). TOM should be suspected when a patient with COM presents facial nerve paralysis. 

This patient presented features of chronic otitis media and the presence of purulent discharge in the middle ear. The patient had a fever along with decreased hearing followed by development of left facial nerve paralysis, intestinal perforation, and right facial nerve paralysis in this sequence. This sort of presentation is unusual and rare. Such a case report depicting such a devastating otologic sequel of tuberculosis as seen in this patient has not been reported in literature.

According to most authors, histological findings, such as granulomas, Langhans giant cells, and caseation necrosis, in association with biomolecular positivity (PCR), represent the cardinal diagnostic elements of TOM (12,15). Whereas, PCR amplification can be used to increase diagnostic accuracy, Ziehl-Nielsen staining should not be considered as a sensitive test, since tuberculous lesions of the ear show low bacterial concentrations (16), which further decline with the use of antibiotic ear drops (e.g., aminoglycoside) (17). Smears of ear drainage with TOM are positive for AFB in 0% to 20% of cases and cultures are positive for Mycobacterium tuberculosis in 5% to 44% of cases (4,18-20). Bacteriological examination of the ear drainage is not very reliable as the presence of other organisms such as Staphylococcus, Pseudomonas, Klebsiella, Proteus, and Streptococus can interfere with the growth of Mycobacterium tuberculosis (20).

CT Scan is reportedly the best imaging technique for TOM (21). Soft tissue attenuation in the entire middle ear cavity, preservation of the mastoid air cells without sclerotic change, and soft tissue extension to, or mucosal thickening of the external auditory canal were more frequent in patients with TOM compared to those having pyogenic chronic otitis with or without cholesteatoma. Erosion of the ossicles and scutum was more frequent in TOM. Temporal bone CT scans of 23 South Korean patients with TOM showed bone destruction that involved the cortex of either the external auditory canal or the outer cortex of the mastoid bone in 26.1% of cases (12). More marked bone destruction involving the base of the skull as well as the mastoid bone was found in only one patient (4.3%).

After confirming the diagnosis of TOM, the treatment of choice is the standard pharmacological treatment used for other forms of tuberculosis. Antituberculosis treatment improves the prognosis for most patients (5). For a complete cure, medical therapy should last for a minimum of nine months and surgical treatment should be added to medical therapy in cases with complications (11).

## Conclusion

TOM is a rare infectious disease of the temporal bone. Because the clinical signs are variable and often different from the disease's classical description, establishing a diagnosis of TOM is difficult. Painless otorrhea, multiple tympanic perforations, early severe hearing loss, abundant granulations, bony necrosis, whitish necrosis tissue of the middle ear mucosa, facial nerve palsy, and malleus handle exposure without mucosa covering are among the features of TOM. Including this disease in the differential diagnosis and considering it will lead to prompt treatment and prevention of serious sequelae.
